# Laparoscopic management of an incarcerated trocar site hernia occurred on the first postoperative day

**DOI:** 10.4103/0972-9941.33278

**Published:** 2007

**Authors:** Vishwanath Golash

**Affiliations:** Department of Surgery, Sultan Qaboos Hospital, P.O.Box: 98, Salalah, Pin Code: 211, Sultanate of Oman

A 41-year-old obese lady presented with severe crampy abdominal pain and vomiting, six hours after her routine laparoscopic cholecystectomy. She was given antiemetic, proton pump blocker and intravenous fluid thinking that she probably had the exacerbation of her acid peptic disease. But she continued to have repeated vomiting, severe colicky abdominal pain and absolute constipation till next day morning. Per abdomen she was tender over her port sites, abdomen was soft and bowel sounds were slightly exaggerated. It was a routine straightforward surgery and it was baffling to know the cause of her acute symptoms. A plain X-ray abdomen was taken which showed the sign of small gut obstruction and a subcutaneous lucent shadow at the 10 mm umbilical port site which was an air filled loop of bowel [Figure 1A]. A computerized tomographic (CT) scan of abdomen with contrast revealed complete obstruction of small bowel and the herniation of a loop of small bowel through the 10 mm umbilical port [Figure 1B]. An urgent relaparoscopy was done which confirmed the findings of CT scan. A loop of small bowel had herniated into the 10 mm umbilical port and was completely obstructed [Figure 2A]. The herniated loop of small bowel was gently reduced back in the abdominal cavity [Figure 2B]. The loop was dusky but recovered in color and had normal peristalsis after few minutes of warm saline irrigation. The port site was closed. She made an uneventful recovery. This is the first port site hernia we had in the immediate postoperative period among the six thousand laparoscopic procedures. The presentation was so dramatic it took us by surprise. This patient was very obese (BMI 57) and long disposable trocars were used. Due to technical difficulty the midline umbilical fascial defect was not closed. It is recommended that the fascia in all port sites larger than 5 mm should be closed under direct laparoscopic vision particularly in obese patients as it is difficult to close them from outside through small skin incisions. It is advisable to remove the ports slowly and under vision because the bowel or omentum may get sucked in giving rise to hernia.

**Figure 1A:**
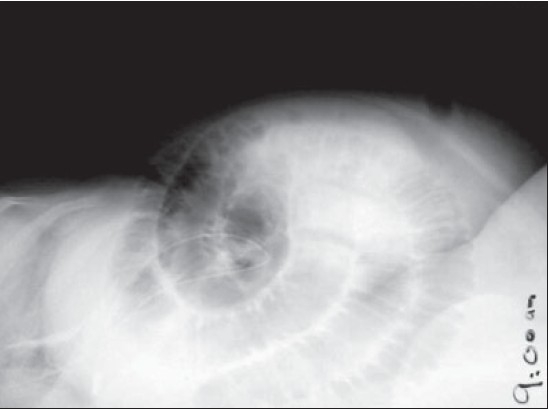
Plain X-ray abdomen showing the air filled loop of small bowel in the umbilical port site

**Figure 1B:**
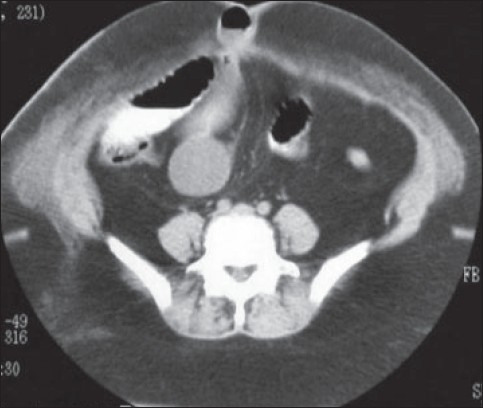
CT scan abdomen showing the complete obstruction of the herniated small bowel loop in the port site

**Figure 2A:**
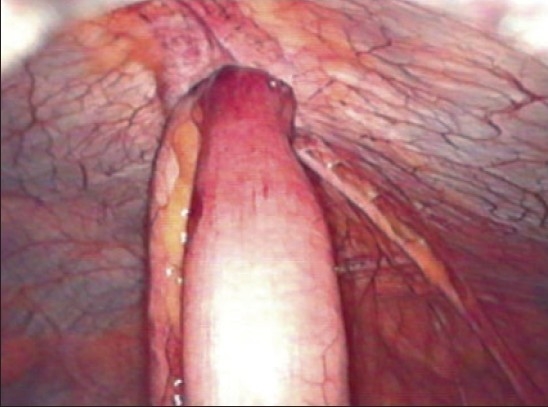
Laparoscopic view of the herniated loop of small bowel

**Figure 2B:**
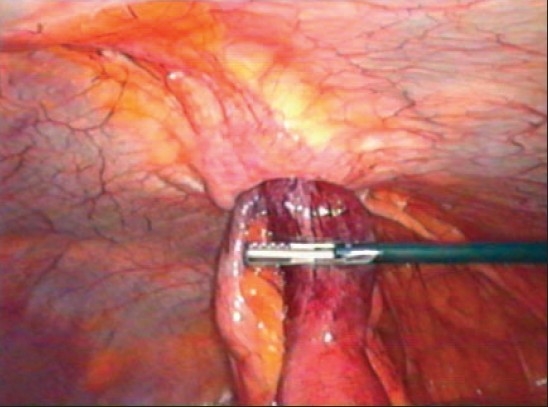
Reducing the herniated loop. Notice the dark dusky discoloration of small bowel

There are several other factors responsible for the development of trocar site hernia. True incidence is not known, as many probably remain asymptomatic. The incidence increases with the number of ports used, larger size ports, dilatation of trocar sites by prolonged and frequent use, removal of gall bladder by dilating the trocar site, repeated withdrawal and reinsertion of ports at the same site, blind introduction of ports, preexisting umbilical hernias, trocar through the umbilical scar, using the port site for the drains, nonclosure or improper closure of fascial defect, breaking of sutures or infection. It usually occurs at midline through large trocar sites and in the majority of cases it is a Richter's type hernia involving the small bowel. Nonbladed trocars probably have lowered the incidence of trocar site hernia. Early diagnosis of trocar site hernia is important to avoid the complications like gangrene and perforation. In our case early CT scan and early surgery prevented from serious complications.

